# Efficient Secretion of Recombinant Proteins from Rice Suspension-Cultured Cells Modulated by the Choice of Signal Peptide

**DOI:** 10.1371/journal.pone.0140812

**Published:** 2015-10-16

**Authors:** Li-Fen Huang, Chia-Chun Tan, Ju-Fang Yeh, Hsin-Yi Liu, Yu-Kuo Liu, Shin-Lon Ho, Chung-An Lu

**Affiliations:** 1 Graduate School of Biotechnology and Bioengineering, Yuan Ze University, 135 Yuan-Tung Road, Taoyuan, Taiwan, ROC; 2 Department of Life Science, National Central University, Taoyuan, Taiwan, ROC; 3 Graduate Institute of Biochemical and Biomedical Engineering, Chang Gung University, Taoyuan, Taiwan, ROC; 4 Department of Agronomy, National Chi-Yi University, Chiayi, Taiwan, ROC; Mayo Clinic Arizona, UNITED STATES

## Abstract

Plant-based expression systems have emerged as a competitive platform in the large-scale production of recombinant proteins. By adding a signal peptide, αAmy3sp, the desired recombinant proteins can be secreted outside transgenic rice cells, making them easy to harvest. In this work, to improve the secretion efficiency of recombinant proteins in rice expression systems, various signal peptides including αAmy3sp, CIN1sp, and 33KDsp have been fused to the N-terminus of green fluorescent protein (GFP) and introduced into rice cells to explore the efficiency of secretion of foreign proteins. 33KDsp had better efficiency than αAmy3sp and CIN1sp for the secretion of GFP from calli and suspension-cultured cells. 33KDsp was further applied for the secretion of mouse granulocyte-macrophage colony-stimulating factor (mGM-CSF) from transgenic rice suspension-cultured cells; approximately 76%–92% of total rice-derived mGM-CSF (rmGM-CSF) was detected in the culture medium. The rmGM-CSF was bioactive and could stimulate the proliferation of a murine myeloblastic leukemia cell line, NSF-60. The extracellular yield of rmGM-CSF reached 31.7 mg/L. Our study indicates that 33KDsp is better at promoting the secretion of recombinant proteins in rice suspension-cultured cell systems than the commonly used αAmy3sp.

## Introduction

Plant-based protein expression systems have been successfully used to produce several recombinant proteins [1–4]. To improve the production of recombinant proteins and reduce the labor and time associated with protein purification steps, various plant-based systems, such as hair roots and suspension-cultured cells which are undifferentiated and actively dividing cells growing in liquid medium, have been considered for the direct secretion of recombinant proteins [5–8]. Most secretory proteins contain N-terminal signal peptides, which lead to the proteins being transported into the endoplasmic reticulum (ER) lumen [9,10] in a signal recognition particle (SRP)-dependent manner *via* SRP receptors [11,12]. Then, the signal peptides are cleaved from the precursor proteins in the ER, and the proteins are packed into small vesicles, budding to the Golgi apparatus, and eventually released to extracellular compartments [13]. A signal peptide is a prerequisite for protein secretion. The secretory proteins are correctly folded and some are further modified in the ER and the Golgi apparatus, such as by N-glycosylation, during the secretory process [14–16]. Signal peptides are fused at the N-terminal end of recombinant proteins to ensure the secretion of the recombinant proteins outside of the cell through this default pathway. As examples of this, a signal peptide derived from the resident ER protein calreticulin was applied in a plant hair root system [17,18]; in addition, an α-amylase signal peptide was used in a rice suspension-cultured cell system [8]. However, it was reported that the recombinant proteins were not secreted efficiently into extracellular space, and a large proportion of recombinant proteins still remained within the cells [19]. This might have been due to the low activity of the applied signal peptide. Influences of signal peptide on protein secretion have been addressed in prokaryote [20], yeast [21] and mammalian cells [22,23]. Although the detailed mechanism is not clear yet for the correlation between signal peptide and protein secretion, one proposed model is that signal peptide plays an important role to lead recombinant protein into the lumen of the ER [24–26], which is one of limiting steps of protein secretion in a default pathway.

The rice αAmy3/RAmy3D signal peptide has been applied to produce recombinant proteins in the medium of rice suspension-cultured cells. Upon coupling with the *αAmy3*/*RAmy3D* promoter, the αAmy3/RAmy3D signal peptide (αAmy3sp) is sufficient to ensure the secretion of recombinant proteins into medium by sugar-starved rice suspension-cultured cells [27–34]. The advantage of this system is that the *αAmy3*/*RAmy3D* promoter is highly inducible and the expressed recombinant protein can be guided extracellularly by αAmy3sp. However, αAmy3sp leads proteins to either extracellular space or to plastids [35]. Signal peptide of another α-amylase, αAmy7/RAmy1A, also drives target proteins into both extracellular space and chloroplasts [36]. Therefore, α-amylase signal peptides including αAmy3sp are not the most appropriate secretion peptides to use in rice suspension-cultured cells for recombinant protein expression.

Granulocyte-macrophage colony-stimulating factor (GM-CSF) is a cytokine that is generated by T cells, macrophages, endothelial cells, and immune-stimulated fibroblasts. GM-CSF exerts biological effects on the immune system, affecting the development and activation of myeloid precursor cells, macrophages, granulocytes, and dendritic cells. Clinical investigation has shown that GM-CSF can be used in a wide range of applications, such as vaccine adjuvant, cancer therapy, and immunotherapy for malignancies [37–40]. Given that GM-CSF has species specificity, mouse GM-CSF (mGM-CSF) is preferentially used in immune- and cancer-related research when mouse is chosen as a model animal.

Most signal peptides consist of 15 to 30 amino acid residues: a few positively charged residues at the N-terminus (n-region), 10–15 hydrophobic amino acid residues in the middle region (h-region), and a signal peptidase cleavage site at the C-terminus (c-region). The c-region usually contains small uncharged amino acid residues, such as glycine or alanine, as the first (-1) and third (-3) amino acid residues upstream of the signal peptidase cleavage site [41]. However, the sequence of amino acids in different signal peptides can vary widely. Therefore, the performance of a signal peptide is not predictable based on its amino acid residue sequence. In this study, to identify an effective signal peptide for application in a recombinant protein production system based on rice suspension-cultured cells, the secretion efficiency of three signal peptides, αAmy3sp, CIN1sp, and 33KDsp, was analyzed in transgenic cell lines by the expression of signal peptide- green fluorescent protein (GFP) fusion proteins. The percentage of GFP secreted extracellularly was higher when fused with 33KDsp than when fused with the other two signal peptides. Furthermore, 33KDsp was applied to produce mGM-CSF, which resulted in a high level of bioactive mGM-CSF being secreted into the culture medium.

## Materials and Methods

### Plant material

Rice (*Oryza sativa L*.) undifferentiated and actively dividing cells, calli, were derived from seeds of the Tainung No. 67 cultivar as described previously [42]. The calli were used for rice transformation, and transgenic calli were maintained on solid Murashige and Skoog (MS) with 3% (w/v) sucrose and 10 μM 2,4-dichlorophenoxyacetic acid (2,4-D). The transgenic calli were subcultured on same medium every 14 days, until calli were homogenized. For the establishment of rice suspension-cultured cells, calli were incubated in liquid MS medium with 3% (w/v) sucrose and 10 μM 2,4-D. The well-grown calli were transferred into liquid MS medium to generate suspension-cultured cells on a reciprocal shaker at 120 rpm and 26°C in the dark. One milliliter of suspension-cultured cells was subcultured in 25 mL of MS medium every 7 days until suspension cultured cells were homogenized for experiments.

### N-terminal protein sequencing

Culture medium of suspension cells was collected and subjected to ammonium sulfate precipitation. Precipitated culture medium proteins (60–80% of ammonium sulfate) were dissolved in a buffer (20 mM Na-acetate, pH 5.2, 10 mM NaCl), and then subjected to dialysis against the same buffer. The culture medium proteins were further purified by fast protein liquid chromatography with Mono S column (GE Healthcare, Waukesha, WI). Collected fractions were examined by 12% SDS-PAGE and expected molecular weight of 33 kDa proteins were pooled and concentrated using a Vivaspin centrifugal concentrator (GE Healthcare). The purified of 33 kDa protein was subjected to N-terminus amino acid microsequencing using protein / peptide sequencer (ABI, Waltham, MA).

### Plasmid construction

The 117 bp, 96 bp, and 102 bp DNA fragments corresponding to Amy3sp, CIN1sp, and 33KDsp, respectively, were amplified by PCR using cDNA prepared from rice suspension cultured cells with specific primers (Table 1). These DNA fragments were inserted into the *Bam*HI and *Eco*RI sites of pBluescript II SK(+) (Stratagene, La Jolla, CA) to generate pBS-Amy3sp, pBS-CIN1sp, and pBS-33KDsp. A GFP DNA fragment was excised from pUGFP [43] with *Bam*HI and *Eco*RI restriction enzymes. pAHC18, which contains maize ubiquitin promoter, was digested with *Bam*HI to remove the luciferase DNA fragment. Each DNA fragment encoding the signal peptide was excised from pBS-Amy3sp, pBS-CIN1sp, or pBS-33KDsp, and subcloned together with the GFP DNA fragment into *Bam*HI-digested pAHC18 plasmid to generate pUAmy3-GFP, pUCIN1-GFP, and pU33KD-GFP. For Agrobacterium-mediated gene transformation into rice genome, each resultant plasmid was digested with *Hin*dIII, and then the DNA fragment containing Ubi promoter–signal peptide–GFP was cloned into the binary vector pCAMBIA 1301, which was obtained from Cambia (www.cambia.org), at the *Hin*dIII site to generate pAUAmy3-GFP, pAUCIN1-GFP, and pAU33KD-GFP.

**Table 1:** Primers used in this study.

Primers	Sequence	Purpose
Amy3F	aatggatccatgaagaacaacagcagcttg	Amy3sp-GFP construction
Amy3R	aatgaattcctcccagttgaaaccctggaa	Amy3sp-GFP construction
CIN1F	aatggatccatggggactcggctcttggcg	CIN1sp-GFP construction
CIN1R	aatgaattcccggtggacgacatgggacgc	CIN1sp-GFP construction
33KDF	aatggatccatggcggcattaagccagctg	33KDsp-GFP and 33KD-mGM-CSF construction
33KDR	aatgaattcgatgggatacgtcgtcgccgc	33KDsp-GFP and 33KD-mGM-CSF construction
mGMCSF-F	aatgaattcatggcacccacccgctcaccc	33KD-mGM-CSF construction; RT-PCR
mGMCSF-R	aatggatcctcatttttggactggtttttt	33KD-mGM-CSF construction; RT-PCR
actinF	ctgatggacaggttatcacc	RT- PCR
actinR	caggtagcaataggtattacag	RT- PCR

ggatcc: *Bam*HI; gaattc: *Eco*RI

To generate the Ubi promoter-33KD signal peptide-mGM-CSF fusion construct, a 381 bp DNA fragment encoding mGM-CSF was amplified by PCR with specific primers (Table 1) by using pENTR-mGM-CSF [33] as the template. The mGM-CSF DNA fragment was cut with *Bam*HI and *Eco*RI, and incubated together with the *Bam*HI and *Eco*RI double-digested 33KDsp DNA fragment to be subcloned into *Bam*HI-treated pAHC18 to generate pU33KD–mGM-CSF. The resultant plasmid was digested with *Hin*dIII and then cloned into the binary vector pCAMBIA 1301 at the same site to generate pAU33kD-mGM-CSF.

### Rice transformation

Four plasmids, pAUAmy3-GFP, pAUCIN1-GFP, pAU33KD-GFP, and pAU33KD-mGM-CSF, were individually introduced into *Agrobacterium tumefaciens* strain EHA105 [44] by electroporation. Rice transformation was performed by *Agrobacterium*-mediated transformation as described previously [42]. Transformed calli were selected on N6 medium with 3% (w/v) sucrose, 10 μM 2,4-D, and 30 mg/L hygromycin.

### Genomic PCR

Genomic DNA was isolated from each putative transgenic callus as described previously [45], and 200 ng of rice genomic DNA were used as a template for the PCR reaction with specific primers to amplify the transgene *GFP* and the endogenous gene *Actin* as an internal control DNA (Table 1). The PCR product was analyzed using 1% agarose gels and stained with ethidium bromide.

### RT-PCR

Total RNA extraction from rice calli and first-strand cDNA synthesis were performed as described as previously [33]. The first-strand cDNA was diluted 50-fold and then subjected to PCR (22–28 reaction cycles) with *GFP*, *αAmy3*, *CIN1*, *33KD*, and *Act1* gene-specific primers (Table 1).

### Protein gel blot analysis

To obtain total intracellular soluble protein, rice calli or suspension-cultured cells were ground to a fine powder in liquid N_2_, and then extracted with protein extraction buffer (100 mM K_2_HPO_4_, pH 7.8, 1 mM EDTA, 7 mM β-mercaptoethanol, 1% (v/v) Triton X-100, and 1% (v/v) glycerol). To obtain total secreted protein released in the liquid medium, the cell culture medium was centrifuged at 18,000 × *g* and 4°C for 15 min in order to remove the cell debris. The protein concentration was determined using Bio-Rad Protein Assay reagent (Bio-Rad, Hercules, CA). Proteins were separated by 12% SDS-PAGE and then blotted onto Hybond-P membrane (GE Healthcare). Commercially available recombinant GFP (Abcam, Cambridge, MA) produced from *E*. *coli* was used as a positive control. Polyclonal rabbit anti-GFP antibody (Abcam) and Lumi-light Western blotting substrate (Roche, Basel, Switzerland) were used to detect the recombinant GFP. For the detection of recombinant mGM-CSF protein, polyclonal rabbit anti-mGM-CSF antibody (Abcam) was used as described previously [33]. To evaluate the proportion of protein that was secreted to the extracellular environments, 0.5 mL of suspension-cultured cells were incubated in 1 mL of MS with 3% sucrose (+S) or without any carbon source (-S) for various periods. The cell culture medium was collected, and the total soluble intracellular protein was isolated from the rice cells in 1 mL of protein extraction buffer. Both cellular soluble proteins and cell culture medium proteins were loaded into gels at 20 μL per lane for Western blot analysis, and the proportion of protein that was secreted was determined as follows: (target protein in medium / target protein in medium + target protein in the cellular protein fraction) × 100%. The Western blot analysis was repeated 3 times and at least 2 biological replicates were performed for each line.

### Fluorometric assays

Total soluble protein was extracted from each transgenic callus or collected from cell culture medium. One hundred microliters of sample were used for GFP fluorescence detection in a Fluoroskan Ascent FL fluorimeter (Thermo, Vantaa, Finland), with excitation at 490 ± 5 nm and emission at 510 ± 5 nm [46]. Fluorescence was normalized using non-transformed cell extract or medium. The fluorometric analyses were performed 3 times and at least 2 biological replicates were applied for each cell line.

### MTT analysis

Reduction of 3-(4,5-dimethylthiazol-2-yl)-2,5-diphenyltetrazolium bromide (MTT) was applied to measure NFS-60 cell viability for the murine myeloblastic leukemia cell line, NFS-60, as previously described [33]. The NFS-60 cells were cultured in medium containing rice-produced mGM-CSF for 3 days. In this assay, purple formazan was detected in a spectrophotometer at 545 nm.

## Results

### The choice of signal peptides for recombinant protein secretion from rice suspension-cultured cells

**Fig 1:**
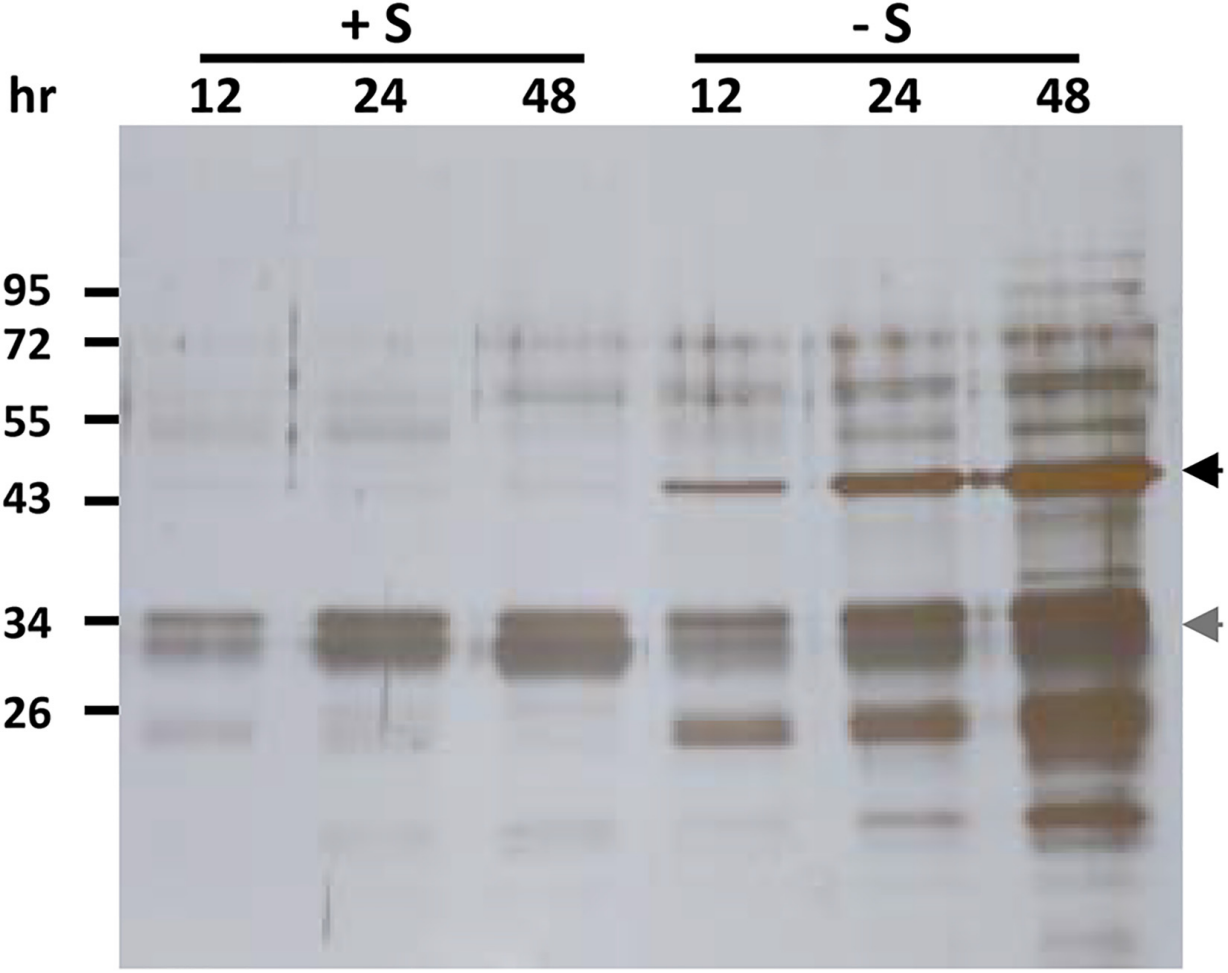
Proteins in medium secreted from suspension-cultured rice cells. One milliliter of rice suspension cells were cultured in 25 mL of sugar-containing medium (+S) or sugar-free medium (-S), for various time periods. Liquid medium samples of 20 μL were obtained, separated by SDS-PAGE, and subjected to silver staining. The location of α-amylase is indicated by the black arrow, and Os33KD protein is indicated by the grey arrow.

**Fig 2:**
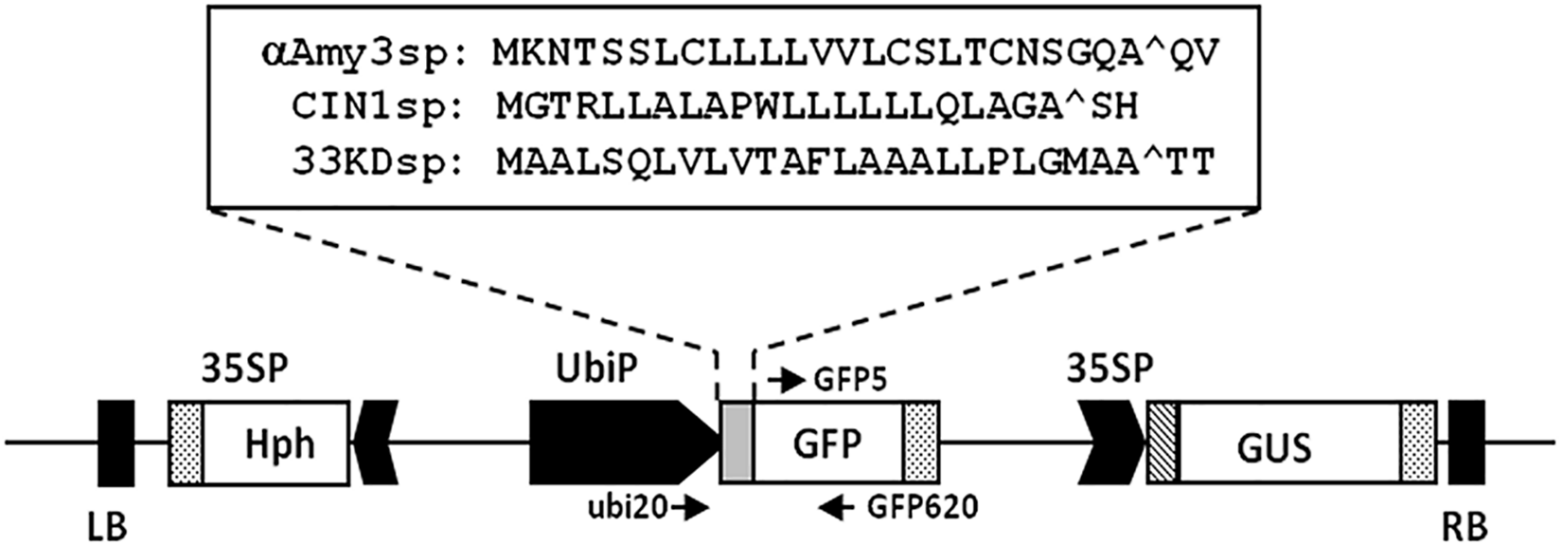
Expression vectors with signal peptides of αAmy3, OsCIN1, or Os33KD used for rice transformation. Various signal peptide cDNAs (αAmy3sp, CIN1sp, or 33KDsp) were fused in-frame upstream of green fluorescent protein (GFP) DNA. The fusion genes were engineered to be located downstream of the maize ubiquitin promoter (UbiP). β-glucuronidase (*GUS*) and the hygromycin-resistance gene (*Hph*) were both driven by a CaMV35S promoter (35SP). Constructs contained the left and right borders (LB, RB) flanking the T-DNA that can be transferred into the plant genome via *Agrobacterium*-mediated transformation. Arrows indicate specific primers used in this study. Amino acid sequences for αAmy3sp, CIN1sp, and 33KDsp are shown in the upper box. Carets indicate predicted signal peptide cleavage sites.

To identify a better signal peptide than rice αAmy3sp, secretory proteins naturally produced by rice suspension cells are good resources to test. The rice suspension cells were cultured in sugar-containing medium or sugar-free medium, for various periods, and the total protein in the medium was analyzed by SDS-PAGE and silver staining. A strong signal at a molecular weight of approximately 33 kDa was observed in both sugar-containing medium and sugar-free medium (Fig 1). The 33 kDa protein was purified by ammonium sulfate precipitation, and then followed by fast protein liquid chromatography. The protein sequence of the corresponding 33 kDa secretory protein was determined, and was identical to the DUF26-like protein/33KD secretory protein (called Os33KD and encoded by *Os04g0659300*), which has been shown as a secretory protein in the culture medium of rice suspension cells [47]. Thus, the signal peptide of Os33KD, called 33KDsp, was selected to compare the secretion efficiency of recombinant proteins with that of αAmy3sp. In addition to 33KDsp, another rice signal peptide was selected, a rice cell wall invertase (OsCIN1, encoded by *Os02g0534400*) which is present in the cell wall and hydrolyzes sucrose to glucose and fructose [48]; this signal peptide was named CIN1sp. The signal peptide regions of αAmy3, Os33KD, and OsCIN1 were predicted using SignalP 3.0 [49], which putatively showed that 25 amino acids at the N-terminus of αAmy3, 25 amino acids at the N-terminus of Os33KD, and 22 amino acids at the N-terminus of OsCIN1 are the signal peptides (Fig 2).

### Comparison of three different signal peptides in terms of the efficiency of recombinant GFP secretion in transgenic rice calli

**Fig 3:**
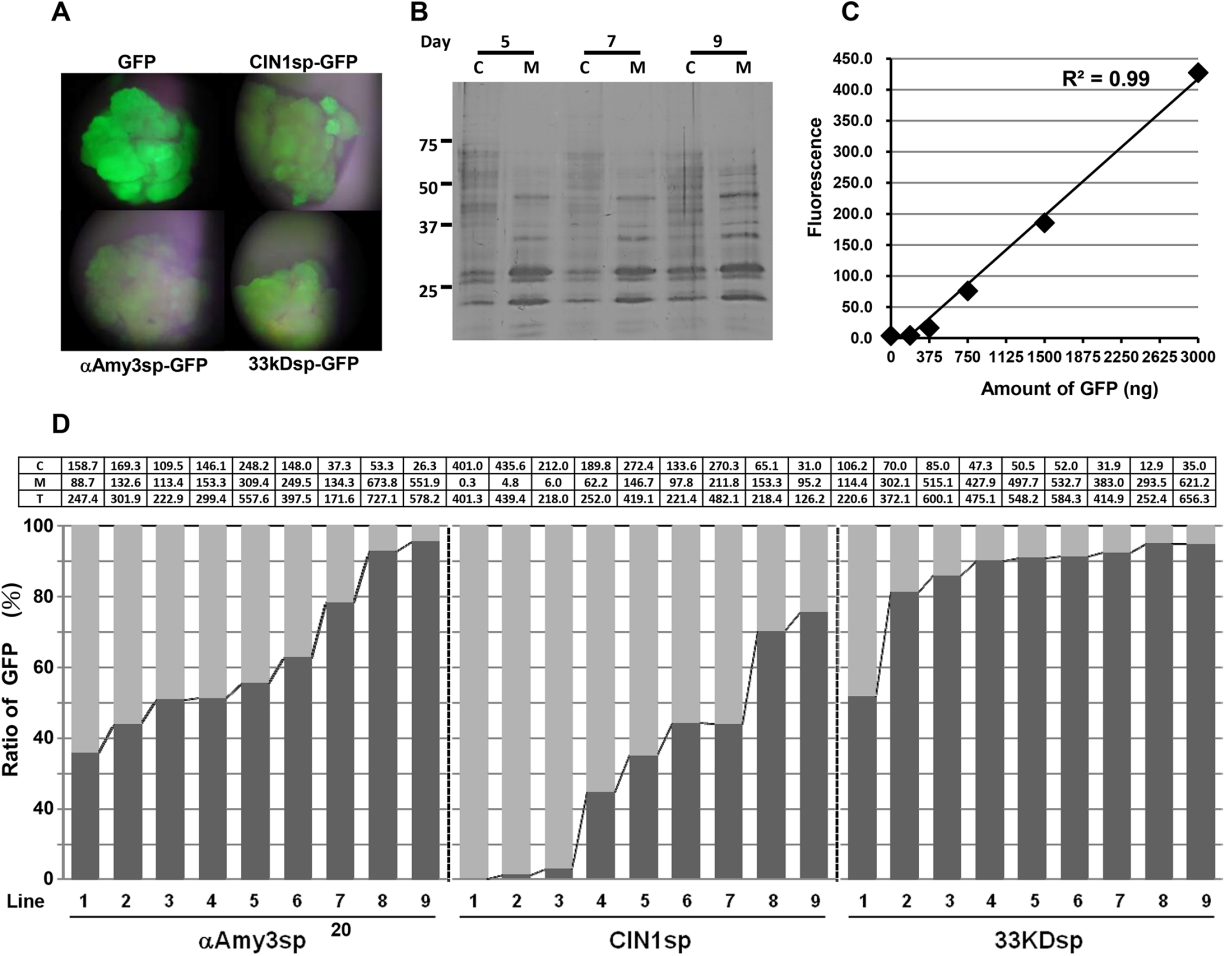
Detection of GFP fluorescence in selected transgenic calli. **A,** Putative transgenic calli harboring the GFP gene were grown on hygromycin selection medium and observed by fluorescence microscopy. **B,** Rice calli (cell volume of 0.5 mL) were cultured in 1 mL of MS with sugar medium for 5, 7 and 9 days. Total cellular soluble proteins were isolated in 1 mL of protein extraction buffer from each of the treated cell cultures. Twenty microliters of total cellular protein (C) and 20 μL of total medium protein (M) were subjected to silver staining. **C,** A standard curve for GFP protein amount and fluorescence intensity. **D,** The ratio of GFP protein in cellular extract and cultured medium among various transgenic cell lines. For each analyzed transgenic line, 0.5 mL rice calli were cultured in 1 mL of MS with sugar medium for 5 days. GFP fluorescence signals from the culture medium and total cellular soluble protein were measured. The upper panel indicated the amount of GFP protein, averaged by three replicated fluorescence detections from two biological repeats. C: amount of cellular GFP proteins (μg); M: total amount of GFP proteins in medium (μg); T: amount of total GFP proteins (μg). The standard deviation from each selected transgenic line was shown in S1 Table. The proportion of medium GFP in each independent line is presented as the GFP fluorescence from total GFP in medium compared with that from total GFP. A dark gray bar indicates medium GFP, while a light gray bar indicates intracellular GFP.

To compare the efficacy of the three rice signal peptides for the efficient secretion of recombinant protein in rice cultured cells, and to reduce influence from other unexpected factors on the secretion efficiency of these signal peptides, ubiquitin (*Ubi*) promoter which drives constitutive gene expression both in sugar feeding and starving rice cultured cells was used to control the expression of reporter gene. Three chimeric genes, *Ubi*::*αAmy3sp-GFP*, *Ubi*::*CIN1sp-GFP*, and *Ubi*::*33KDsp-GFP*, containing αAmy3sp, CIN1sp, and 33KDsp, respectively, were generated (Fig 2). These expression cassettes were introduced individually into the rice genome *via Agrobacterium*-mediated transformation. Several transgenic lines were obtained for each signal peptide, and GFP fluorescence was observed as shown in Fig 3A. Nine transgenic lines of each construct were randomly selected to detect GFP levels. We previously demonstrated that the cell viability of suspension-cultured rice cells in sugar containing medium can be maintained 90% at least for 10 days [26]. We further confirmed the result by protein gel analysis, and the band patterns of intracellular soluble proteins were obviously different from cultured medium proteins at 5, 7 and 9 days (Fig 3B). Therefore, the transgenic calli were cultured in MS liquid medium for 5 days, and both cultured calli and medium were collected to prevent artifacts caused by cell lysis. To quantify the level of fluorescence of GFP, a standard curve was used with a concentration range from 0.1 to 3 μg/mL purified recombinant GFP protein (Fig 3C). For each independent cell line, the levels of recombinant GFP proteins in the culture medium and in cellular extracts were measured (Fig 3D; S1 Table), and the proportion of GFP protein secreted was determined by dividing the amount of GFP in the medium by the total GFP. As shown in Fig 3D, wide ranges of intracellular to medium GFP ratios were displayed among the selected lines. For *αAmy3sp-GFP* transgenic cell lines, 37%–95% of total GFP was detected in the medium. For *CIN1spGFP* transgenic cell lines, 0%–75% of total GFP was detected in the medium. However, for *33KDspGFP* transgenic cell lines, 52%–95% of total GFP was detected in the medium, and in the majority of these selected lines, more than 80% of total GFP was present in the culture medium. This result showed that the highest proportion of medium GFP was achieved using 33KDspGFP-containing calli, followed by αAmy3spGFP and then CIN1spGFP, suggesting that the secretion efficiency of 33KDsp is higher than that of αAmy3sp and CIN1sp.

### Efficiency of three signal peptides for the secretion of recombinant GFP proteins in transgenic suspension-cultured rice cells

**Fig 4:**
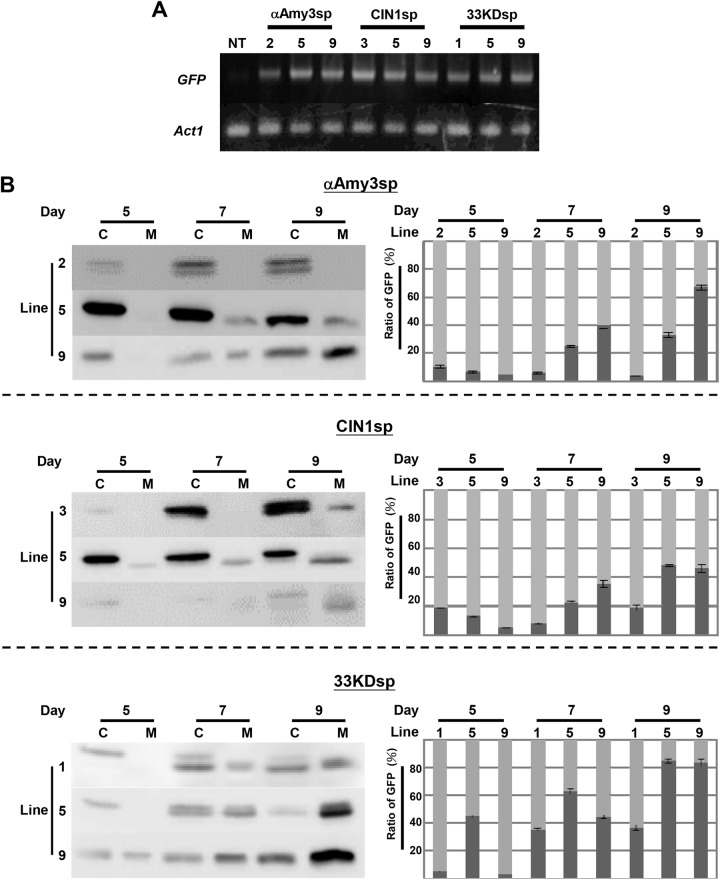
Gene expression of GFP in transgenic rice suspension cell cultures. **A,** Detection of GFP mRNA in non-transformed (NT) and selected transgenic rice suspension-cultured cells, including αAmy3sp-2, -5, and -9; CIN1sp-3, -5, and -9; and 33KDsp-1, -5, and -9. Total RNAs were isolated from NT and transgenic rice suspension-cultured cells, and then subjected to RT-PCR with specific *GFP* primers. The rice *Act1* mRNA was used as an internal control. **B,** The proportion of medium GFP protein in various transgenic suspension cell lines. Total soluble proteins were extracted from each cell line, with a cell volume of 0.5 mL, in 1 mL of extraction buffer. A total of 1 mL of culture medium for each line was collected. Twenty microliters of total cellular protein (C) and 20 μL of total extracellular protein (M) were subjected to Western blot analysis using GFP antibodies. GFP proteins within cells and medium were detected, and compared with the total amount of GFP protein. The proportion of medium GFP for each independent cell line is presented as the total GFP in medium compared with the total GFP. A dark gray bar indicates medium GFP, while a light gray bar indicates cellular GFP. Error bars indicate the standard deviation of three Western blot replicates from two biological repeats.

To evaluate the secretion efficiency of the three signal peptides further, three independent suspension-cultured cell lines for each signal peptide were established. The mRNA levels of *GFP* in the various suspension cell lines were similar, except for line 2 of *αAmy3sp-GFP*, which had a slightly lower level, and line 3 of *CIN1sp-GFP*, which had a higher level than the other cell lines (Fig 4A). Each selected rice suspension-cultured cell line was cultured in MS medium for 5, 7, and 9 days, after which total cellular soluble protein and culture medium protein were obtained. The relative levels of GFP was detected by Western blot analysis with GFP antibodies, and quantified using Gel-Pro Analyzer (Fig 4B). The GFP proteins were detected in both intracellular and medium total soluble proteins of most suspension cell lines (Fig 4B). Compared with the *αAmy3sp-GFP* suspension cell lines and the *CIN1sp-GFP* suspension cell lines, the *33KDsp-GFP* suspension cell lines accumulated more recombinant GFP proteins in the culture medium, regardless of the duration of the culture period (Fig 4B).

**Fig 5:**
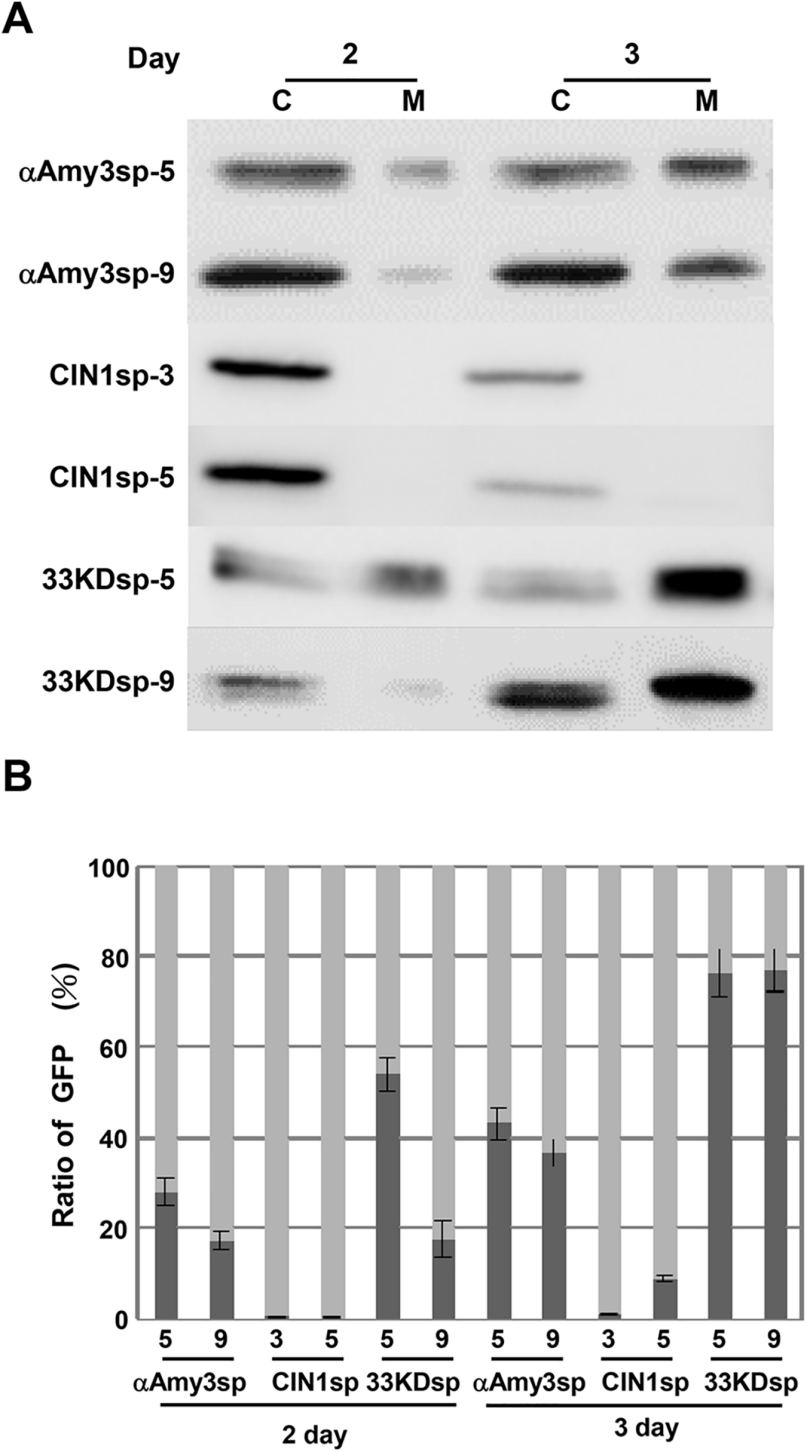
Detection of GFP protein in sugar-starved transgenic rice suspension cell cultures. **A,** Rice suspension cells (cell volume of 0.5 mL) were cultured in 1 mL of sugar-free MS medium for 2 and 3 days. Total cellular soluble proteins were isolated in 1 mL of protein extraction buffer from each of the treated cell cultures. Twenty microliters of total cellular protein (C) and 20 μL of total medium protein (M) were subjected to Western blot analysis using GFP antibodies. **B,** The proportion of medium GFP protein in various transgenic suspension cell lines. The amount of GFP within cells and medium was detected, and converted to the total amount of GFP protein. The proportion of medium GFP in each independent line is presented as the total GFP in the medium compared with the total GFP. A dark gray bar indicates medium GFP, while a light gray bar indicates cellular GFP. Error bars indicate the standard deviation of three Western blot replicates from two biological repeats.

A recombinant protein expression platform in rice suspension cells has been established using a rice α-amylase gene promoter, *αAmy3*p, which drives the production of recombinant protein in sugar-free medium [29]. To evaluate the secretion efficiency of the three signal peptides in sugar-free growth conditions, two suspension-cultured cell lines per signal peptide were incubated in sugar-free medium for 2 and 3 days. As shown in Fig 5, in 2-day-cultured medium, there was substantially more accumulation of recombinant GFP proteins for the *33KDsp-GFP* lines than for the *αAmy3sp-GFP* lines. When suspension-cultured cells were starved of sugar for 3 days, GFP proteins further accumulated in the culture medium, and even more GFP was secreted by the *33KDsp-GFP* cell lines than by *αAmy3sp-GFP*. These results indicate that 33KDsp has higher secretion efficiency than αAmy3sp, and can be applied in a well-developed recombinant protein production platform using sugar-starved rice suspension-cultured cells.

### Production of bioactive mGM-CSF in the medium of rice suspension-cultured cells using the 33KD signal peptide

**Fig 6:**
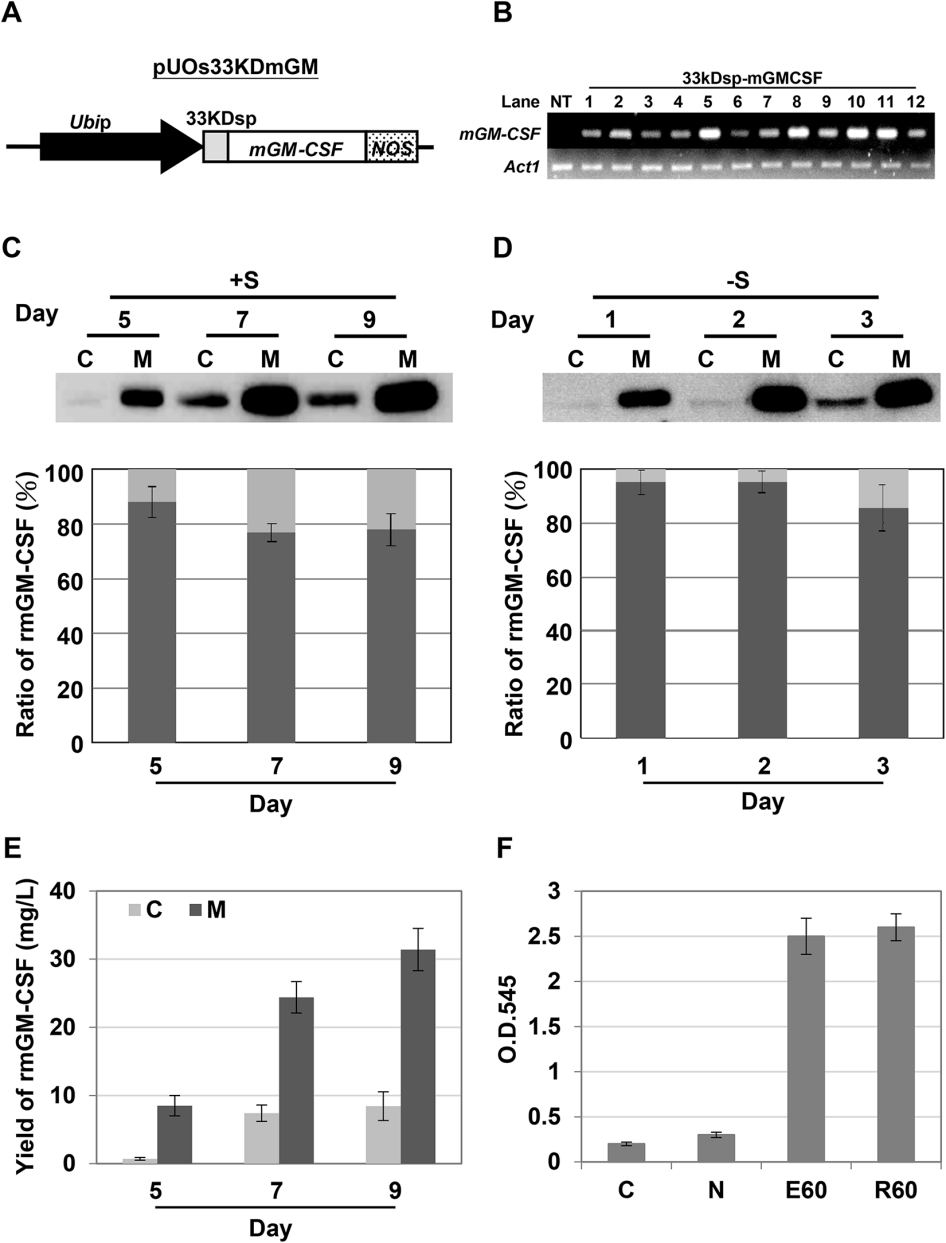
Production of secretory recombinant mGM-CSF in rice cells using the 33KD signal peptide. **A,** Expression vector containing a 33KD signal peptide fused with mGM-CSF chimeric gene. The 33kDsp-mGM-CSF fusion gene was engineered to be located downstream of the maize ubiquitin promoter (UbiP). **B**, Detection of mGM-CSF mRNA in non-transformed (NT) and selected 33KDsp-mGMCSF putative transgenic rice calli. Total RNAs were isolated from NT and transgenic rice calli, and then subjected to RT-PCR with specific mGM-CSF primers. The rice *Act1* mRNA was used as an internal control. **C & D,** The proportion of medium rice-derived mGM-CSF (rmGM-CSF) protein in transgenic suspension cell line 10. Suspension cells with cell volume of 0.5 mL were cultured in 1 mL of medium with (C) or without sugar (D). Total soluble proteins were isolated from the suspension cells in 1 mL of protein extraction buffer, and 1 mL of culture medium was collected at various time periods as indicated. Twenty microliters of total cellular proteins (C) and 20 μL of total extracellular proteins (M) were subjected to Western blot analysis using mGM-CSF antibodies. The signals for rmGM-CSF within cells and medium were detected and compared with the total amount of rmGM-CSF protein. The proportion of medium rmGM-CSF is presented as the total rmGM-CSF in the medium compared with the total rmGM-CSF. A dark gray bar indicates medium rmGM-CSF, while a light gray bar indicates cellular rmGM-CSF. Error bars indicate the standard deviation of three Western blot replicates from two biological repeats. **E,** Production of mGM-CSF in transgenic rice suspension cells cultured in sugar-containing medium. The amount of recombinant mGM-CSF produced was measured in culture medium and rice suspension cells at 5, 7, and 9 days. Error bars indicate the standard deviation of three Western blot replicates from two biological repeats. **F,** Analysis of the biological activity of secreted rmGM-CSF from rice suspension cell culture medium at day 9. A murine hematopoietic cell line (NFS-60) was incubated with 60 ng/mL rice-derived mGM-CSF (R60). Commercial mGM-CSF derived from *E*. *coli* cells (E60; 60 ng/mL) was used as a reference standard. Both sucrose-free MS medium (C) and non-transformed rice culture medium (N) were used as negative controls. The proliferation of NFS-60 cells was measured with the MTT assay system. The error bar represents the standard deviation from triplicate cultures.

To examine whether 33KDsp can be applied for the efficient secretion of other valuable recombinant proteins, mGM-CSF was fused downstream of 33KDsp and expression was driven by the Ubi promoter (Fig 6A). Through the *Agrobacterium*-mediated transformation, several transgenic cell lines were obtained, and line 10 with a high level of *mGM-CSF* mRNA was selected to establish the rice suspension-cultured cell line (Fig 6B). The suspension-cultured cells were incubated in either sugar-containing medium for 5, 7, and 9 days or sugar-free medium for 1, 2, and 3 days, and then subjected to rmGM-CSF monitoring of the total soluble intracellular and medium proteins. As shown in Fig 6C, rice-derived mGM-CSF (rmGM-CSF) was detectable in both cell extract and culture medium, and its level increased with time in sugar-containing medium (Fig 6C). The rmGM-CSF was predominantly present in culture medium, at around 76%–92% of total rmGM-CSF protein (Fig 6C). The yields of rmGM-CSF were 8.5, 24.4, and 31.7 mg/L in sugar-containing medium cultured for 5, 7, and 9 days, respectively (Fig 6E). When the rmGM-CSF production by suspension cell line 10 was monitored upon culture in sugar-free medium for 1, 2, and 3 days, the protein was predominantly detected in the sugar-free culture medium, and the percentage of rmGM-CSF that was secreted reached 90% of the total amount produced (Fig 6D).

The biological activity of rice-derived rmGM-CSF secreted due to the 33KD signal peptide was examined with a colorimetric microassay using murine myeloblastic leukemia NFS-60 cells. The NFS-60 cells were cultured with rice suspension cell- or *E*. *coli*-derived recombinant mGM-CSF for 3 days. Cell viability was determined using the MTT assay system. Neither sucrose-free MS medium (Fig 6F, column C) nor non-transformed rice culture medium (Fig 6F, column N) could proliferate the NSF-60 cells. In contrast, the viability of NFS-60 cells was increased significantly in the presence of mGM-CSF (Fig 6F). The activities of the two types of mGM-CSF: recombinant mGM-CSF produced from *E*. *coli* (commercial product as a positive control) or that from rice suspension cells, were similar in terms of the viability of NFS-60 cells that they enabled (Fig 6F).

## Discussion

The production of recombinant proteins in cell cultured medium have been conducted in several recombinant protein expression systems. In addition, recombinant proteins that require post-translational modification in order to retain bioactivity or stability can be modified through a series of enzymes located in the ER and the Golgi apparatus before secretion. Rice suspension-cultured cells, used in conjunction with an αAmy3 signal peptide, have been used to successfully produce several recombinant proteins in culture medium. However, the secretion efficiency of the αAmy3 signal peptide in rice suspension cells had not been clarified. In this study, we compared the secretion efficiency of three rice signal peptides, αAmy3sp, CIN1sp, and 33KDsp, in transgenic rice cell lines that expressed signal peptide fused with GFP. The amount of medium GFP as a proportion of the total GFP was used as an indicator of the secretion efficiency. Our results indicated that 33KDsp was more efficient than αAmy3sp and CIN1sp for the secretion of GFP from both rice calli and suspension-cultured cells. 33KDsp was also used to secrete mGM-CSF from transgenic rice suspension-cultured cells, and 76%–92% of the mGM-CSF was detected in the culture medium.

The general features of signal peptides that increase the efficiency of secretion in rice cells are unclear. In this study, although GFP could be secreted out of rice cells, intracellular GFP was still detectable. Thus, the studied signal peptides were not able to cause all GFP to be secreted. However, we cannot rule out possibility that secreted GFP was retained between outside of the cell membrane and the cell wall matrix or in the intracellular space of the cell aggregates.

Plant cell wall porosity has been measured around 3.5–9.2 nm by different measurement methods, thus proteins smaller than 30–50 kDa can easily pass through a plant cell wall [50]. The molecular weight of GFP is 27 kDa, so it can be secreted into cultured medium as expected. In this study, two forms of GFP, 30 kDa and 27 kDa, were identified in Western blot analysis. The 27 kDa GFP was the same as that of the medium GFP. Therefore, we assume that the 30 kDa GFP was due to a failure of the signal peptide to be cleaved. This was supported by the fact that the amount of the 30 kDa GFP was inversely correlated with the secretion efficiency. For example, the 30 kDa GFP was accumulated more in the *CIN1sp*-*GFP* transgenic cell lines that had a low level of extracellular GFP. In contrast, a low level of the 30 kDa GFP was detected in the *33KDsp*-*GFP* transgenic cell lines that had high secretion efficiency. These observations suggest that the cleavage of the signal peptide is an important step for protein secretion by rice suspension cells. The three signal peptides studied here all contain classic cleavage sites in the c-region, and the first (-1) and third (-3) amino acids upstream of the cleavage site are small and uncharged amino acids; therefore, primary sequence are not able to offer cleavage efficiency of these signal peptides.

Low affinity between signal peptide and SRP may lead to a failure of secretion in a default pathway. The hydrophobic h-region of the signal peptide forms an α-helix that is bound by the SRP [51] and this step is necessary for secretory proteins to translocate into the lumen of the ER. Then, the signal peptides are removed from secretory proteins in the secretory pathway. On the basis of previous studies on hydrophobic residues of the interleukin-2 signal peptide [26] and the *Metridia* luciferase signal peptide [22] in mammalian cells, secretion efficiency can be improved by increasing the hydrophobicity. The three signal peptides studied here all contain an h-region, and the highest hydrophobicity is exhibited by Amy3sp, followed by CIN1sp and 33KDsp. However, 33KDsp had the highest secretion efficiency, so high hydrophobicity of the h-region was clearly not the primary determinant of this. Instead, it is possible that 33KDsp has specific amino acid residues in the h-region that are important for association with the SRP.

The levels of GFP mRNA did not differ markedly among the selected transgenic cell lines harboring various signal peptides fused to GFP. However, the amounts and secretion ratios of GFP did differ markedly among the different lines, even for the same signal peptide. There is no clear correlation among GFP mRNAs, cellular GFP proteins and secreted GFP proteins, and complicated dynamic cellular activities may interfere the relationship between cellular and secreted GFP. In addition, individual differences among independent transgenic lines also increase the variation. For example, it is common to observe most transgenic lines have various expression levels of transgene, even harboring the same expression cassette. Our results also showed that CIN1sp was ineffective in the rice suspension cell expression system. Both αAmy3sp and 33KDsp were better signal peptides than CIN1sp in our studies, and both *αAmy3* and *Os33KD* gene expression were abundant in the rice cell suspension cultured medium. In contrast, gene expression of *OsCIN1* was strong in leaves [52] and weak in calli [53]. Thus, it would be an interesting topic to study regulatory roles of signal peptides on protein secretion in different types of cells.

In this study, we used the maize *Ubi* promoter, a constitutive expression promoter, to examine the efficiency of secretion promoted by signal peptides in rice suspension-cultured cells. The *Ubi* promoter with 33KDsp was further applied for mGM-CSF production. The bioactive form of rice recombinant mGM-CSF was detected in the culture medium and the maximum yield reached 31.7 mg/L. Our study indicates that application of the constitutive *Ubi* promoter with 33KDsp is recommended in protein expression systems with rice suspension-cultured cells instead of the sugar starvation-inducible *αAmy3* promoter with αAmy3sp.

## Supporting Information

S1 Table Cellular, and medium GFP contents (μg) in transgenic lines studied in Fig 3. (DOCX)
