# Supplementary material for: Efficient Secretion of Recombinant Proteins from Rice Suspension-Cultured Cells Modulated by the Choice of Signal Peptide
Source: PLoS One. 2015 Oct 16;10(10):e0140812. doi: 10.1371/journal.pone.0140812 (PMC4608814; doi:10.1371/journal.pone.0140812)
Supplement: S1 Table — (DOCX) [file pone.0140812.s001.docx]

| **S1 Table. Cellular, and medium GFP contents (μg) in transgenic lines studied in Figure 3.** | | | | | | | | | |
| --- | --- | --- | --- | --- | --- | --- | --- | --- | --- |
| αAmy3sp |  | | | | | | | | |
|  | Line No. | | | | | | | | |
|  | 1 | 2 | 3 | 4 | 5 | 6 | 7 | 8 | 9 |
| Cell | 158.7±3.8 | 169.3±6.1 | 109.5±6.3 | 146.1±10.6 | 248.2±9.5 | 148.0±8,4 | 37.3±4.6 | 53.3±8.4 | 26.3±6.2 |
| Medium | 88.7±6.6 | 132.6±7.5 | 113.4±12.8 | 153.3±12.3 | 309.4±11.9 | 249.5±23.8 | 134.3±9.0 | 673.8±16.8 | 551.9±17.5 |
| Total | 247.4 | 301.9 | 222.9 | 299.4 | 557.6 | 397.5 | 171.6 | 727.1 | 578.2 |
| CIN1sp |  | | | | | | | | |
|  | Line No. | | | | | | | | |
|  | 1 | 2 | 3 | 4 | 5 | 6 | 7 | 8 | 9 |
| Cell | 401.0±15.4 | 435.6±24.9 | 212.0±12.6 | 189.8±12.5 | 272.4±14.6 | 133.6±10.7 | 270.3±13.6 | 65.1±7.7 | 31.0±5.1 |
| Medium | 0.3±0.1 | 4.8±5.2 | 6.0±7.3 | 62.2±4.8 | 146.7±8.9 | 97.8±4.2 | 211.8±11.1 | 153.3±6.2 | 95.2±13.5 |
| Total | 401.3 | 439.4 | 218.0 | 252.0 | 419.1 | 221.4 | 482.1 | 218.4 | 126.2 |
| 33KDsp |  | | | | | | | | |
|  | Line No. | | | | | | | | |
|  | 1 | 2 | 3 | 4 | 5 | 6 | 7 | 8 | 9 |
| Cell | 106.2±8.2 | 70.0±3.7 | 85.0±8.1 | 47.3±5.5 | 50.5±4.2 | 52.0±6.7 | 31.9±3.8 | 12.9±2.1 | 35.0±2.4 |
| Medium | 114.4±8.4 | 302.1±13.1 | 515.1±18.5 | 427.9±15.7 | 497.7±22.0 | 532.7±31.2 | 383.0±20.4 | 293.5±17.8 | 621.2±23.9 |
| Total | 220.6 | 372.1 | 600.1 | 475.1 | 548.2 | 584.3 | 414.9 | 252.4 | 656.3 |
| Error bars indicate the SDs of three independent biological replicates. | | | | | | | | | |
